# Neuregulin and Dopamine D4 Receptors Contribute Independently to Depotentiation of Schaffer Collateral LTP by Temperoammonic Path Stimulation

**DOI:** 10.1523/ENEURO.0176-17.2017

**Published:** 2017-08-21

**Authors:** Yukitoshi Izumi, Charles F. Zorumski

**Affiliations:** 1 Department of Psychiatry, Washington University School of Medicine, St. Louis, MO 63110; 2 Department of Neuroscience, Washington University School of Medicine, St. Louis, MO 63110; 3 Taylor Institute for Innovative Psychiatric Research, Washington University School of Medicine, St. Louis, MO 63110; 4 Center for Brain Research in Mood Disorders, Washington University School of Medicine, St. Louis, MO 63110

**Keywords:** adenosine, endocannabinoids, ErbB, GABA, hippocampus, perforant path

## Abstract

Prior studies have found that dopamine (DA), acting at D4 receptors, and neuregulin (NRG), likely acting at ErbB4 receptors, are involved in a form of depotentiation of long-term potentiation (LTP) at Schaffer collateral (SC) synapses in the hippocampus. Furthermore, DA and NRG actions are intertwined in that NRG induces DA release. We previously found that low-frequency stimulation (LFS) of temperoammonic (TA) inputs to area CA1 also depotentiates previously established SC LTP through a complex signaling pathway involving endocannabinoids, GABA, adenosine, and mitogen-activated protein kinases (MAPKs), but not glutamate. In the present studies, we found that TA-induced SC depotentiation in hippocampal slices from Sprague-Dawley albino rats also involves activation of both D4 receptors and NRG-activated ErbB receptors, but that the roles of these two modulator systems are independent with D4 receptor antagonism failing to alter chemical depotentiation by NRG1β. Furthermore, a selective D4 receptor agonist was unable to depotentiate SC LTP when administered alone, suggesting that D4 receptor activation is necessary but not sufficient for TA-induced SC depotentiation. Chemical depotentiation by NRG1β was inhibited by a Pan-ErbB antagonist and by picrotoxin (PTX), an antagonist of GABA-A receptors (GABA_A_Rs), indicating that NRG likely promotes SC depotentiation via effects on GABA and interneurons. These findings have implications for understanding the role of DA and NRG in cognitive dysfunction associated with neuropsychiatric illnesses.

## Significance Statement

Low frequency activation of temperoammonic (TA) inputs to stratum lacunosum moleculare (SLM) of hippocampal area CA1 can heterosynaptically depotentiate previously established long-term potentiation (LTP) of Schaffer collateral (SC) synapses. TA-induced depotentiation involves complex signaling via endocannabinoids, GABA and adenosine. Other studies indicate that SC depotentiation can involve activation of dopamine (DA) D4 receptors following DA release mediated by neuregulin-1 (NRG1). In the present studies, we find that both D4 receptors and NRG1 contribute to TA-induced SC depotentiation but do so independently. These findings have implications for understanding cognitive defects associated with psychiatric disorders.

## Introduction

Hippocampal synapses operate over a dynamic range of efficacy and are subject to both short- and long-term forms of plasticity, including long-term potentiation (LTP) and long-term depression (LTD), leading candidates as synaptic memory mechanisms (Malenka and Bear, 2004; Kandel et al., 2014, Nicoll, 2017). Because there are limits on the degree to which hippocampal synapses can potentiate or depress, there is interest in determining how synapses reset to baseline following the induction of stable synaptic plasticity. This interest is compounded by the fact that the hippocampus is a short-term, limited capacity storage system. Potential mechanisms for synaptic resetting include homeostatic plasticity, in which synapses adjust to changes in activity over time (Turrigiano, 2011), and homosynaptic resetting, in which the same synapses that are altered instruct their own resetting (Fujii et al., 1991; Bashir and Collingridge, 1994).

We have been interested in determining whether extra-hippocampal inputs can instruct Schaffer collateral (SC) synapses to reset heterosynaptically following successful induction of stable LTP. We have found that low-frequency stimulation (LFS) of temperoammonic (TA) inputs to stratum lacunosum moleculare (SLM) in area CA1 can induce depotentiation (LTP-D) of SC synapses without persistently altering baseline SC transmission or the ability of SC synapses to undergo subsequent LTP after resetting (Izumi and Zorumski, 2008). TA-induced LTP-D involves complex signaling including activation of GABA-A receptors (GABA_A_Rs), cannabinoid-1 receptors (CB1Rs) and adenosine A1 receptors (A1Rs), and activation of mitogen-activated protein kinase (MAPK) signaling, including extracellular signal-related kinase 1/2 (ERK1/2) and p38 MAPK (Izumi and Zorumski, 2016).

Surprisingly, TA-induced LTP-D does not involve activation of AMPA-type glutamate receptors, NMDA receptors, metabotropic glutamate receptors, or L-type voltage-activated calcium channels (Izumi and Zorumski, 2008, 2016). These latter observations led us to consider the role of other inputs in SLM. Besides direct glutamatergic inputs and long-range GABAergic inputs from entorhinal cortex (Basu et al., 2016), SLM receives input from neuromodulatory systems, including monoamines (Swanson and Hartman, 1975), and dopamine (DA) has previously been shown to dampen direct TA glutamatergic inputs from entorhinal cortex but not SC pathway responses (Otmakhova and Lisman, 1998). Other studies, however, have shown that activation of DA D4 receptors (D4Rs) can drive a form of SC depotentiation when activated within 30 min of LTP induction (Kwon et al., 2008). This form of LTP-D is induced by neuregulin-1 (NRG1) and involves activation of ErbB receptors, which in turn enhance DA release (Kwon et al., 2005, 2008). Similarly, homosynaptic activation of SC inputs by theta pulse stimulation can reverse SC LTP via D4Rs when administered shortly after LTP induction (Kwon et al., 2008). Other studies indicate that low frequency activation of DA fibers in the hippocampus can stimulate D4Rs to dampen SC responses via activation of parvalbumin positive interneurons (Rosen et al., 2015). Based on these observations, we examined the roles of DA and NRG in TA-induced LTP-D.

## Materials and Methods

### Hippocampal slices

Protocols for animal use were approved by the Washington University Animal Studies Committee in accordance with national and international guidelines. Hippocampal slices were prepared from the septal (dorsal) hippocampal region of postnatal day (P)28-P32 Sprague Dawley albino rats using previously described methods (Izumi and Zorumski, 2008; Tokuda et al., 2010). Pregnant female rats were purchased from Charles River (Crl:CD(SD), RRID:RGD_734476); male offspring were raised to age 28-32 d in an approved animal care facility. On the day of experiments, rats were anesthetized with isoflurane, decapitated, and hippocampi were dissected. Isolated hippocampi were placed in ice-cold artificial CSF (ACSF) containing 124 mM NaCl, 5 mM KCl, 2 mM MgSO_4_, 2 mM CaCl_2_, 1.25 mM NaH_2_PO_4_, 22 mM NaHCO_3_, 10 mM glucose, bubbled with 95% O_2_-5% CO_2_ at 4-6°C, and cut into 450-μm slices using a rotary tissue slicer. The slices were cut to include a significant portion of entorhinal cortex to keep TA inputs to SLM in the CA1 region intact to the extent possible (Izumi and Zorumski, 2008, 2016). In the present experiments we did not monitor field potentials in SLM directly because these potentials reflect a combination of several inputs from entorhinal cortex and other regions (Basu et al., 2016; Swanson and Hartman, 1975), although prior studies indicate that repeated LFS of the TA pathway, akin to what we use in this study, produces LTD of these inputs (Dvorak-Carbone and Schuman, 1999). After preparation, slices were allowed to recover from dissection in an incubation chamber containing gassed ACSF for 1 hr at 30°C before experiments.

### Hippocampal slice physiology

At the time of study, slices were transferred individually to a submersion-recording chamber. Experiments were done at 30°C with continuous ACSF perfusion at 2 ml/min. Extracellular recordings were obtained from the apical dendritic layer (stratum radiatum) of the CA1 region for analysis of EPSPs using glass electrodes filled with 2 M NaCl (5-10 MΩ resistance).

EPSPs were evoked using 0.1-ms constant current pulses through a bipolar stimulating electrode in the SC pathway. A second stimulating electrode was placed in the TA pathway to activate inputs to CA1 in SLM. A control input-output curve was obtained to determine stimulus intensities for subsequent studies. Responses were monitored by applying single stimuli to the SC pathway every 60 s at half maximal intensity. After establishing a stable baseline for at least 10 min, SC LTP was induced by a single 100 Hz × 1 s high-frequency stimulus (HFS) using the same intensity stimulus. Input-output curves were repeated 60 min following tetanic stimulation. TA stimulation to induce SC depotentiation was administered as a 1 Hz × 15 min LFS at half maximal intensity based on prior results (Izumi and Zorumski, 2008, 2016).

### Materials and Methods

Chemicals and pharmacological agents were obtained from Tocris or Sigma (St. Louis MO). NRG1β was obtained from R&D Systems. Concentrations of all agents used in this study (agonists and antagonists) and durations of exposure were based on published literature and, more specifically, on the lack of effect on baseline SC transmission under the conditions of our experiments. The exception to this was picrotoxin (PTX), a GABA_A_R antagonist that induced changes in basal EPSPs even at the low concentration (1 μM) used for the experiments in Figure 5. Because of the changes in basal transmission, PTX was washed on at the initiation of the recordings and included in ACSF for the duration of these experiments.

### Statistical analysis

**Figure 1.**
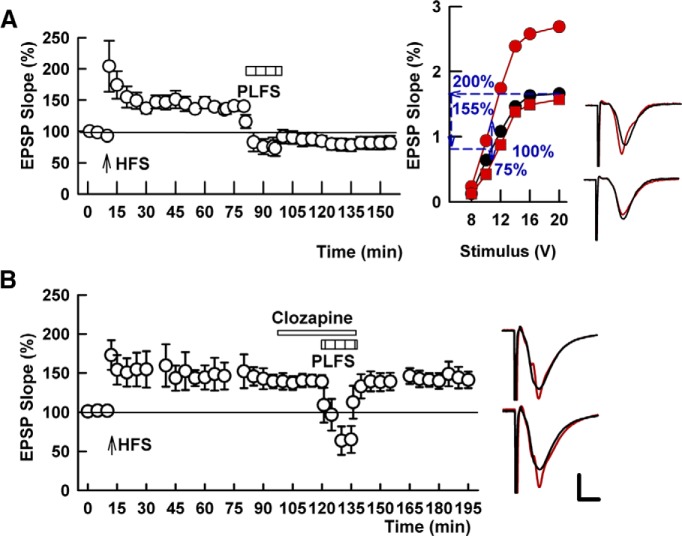
TA-induced LTP-D is blocked by the DA receptor antagonist, clozapine. ***A***, The left graph shows the time course of changes in EPSPs following SC HFS (arrow) and depotentiation by TA/perforant path LFS (PLFS, bar). Note that data in ***A*** include control slices done in our prior manuscript (Izumi and Zorumski, 2016) with additional slices added. The right graph in ***A*** depicts an analysis from a single slice based on changes in the IO curve. Black circles are baseline IO results, while red circles depict changes 60 min after SC HFS, and red squares show reversal of LTP following PLFS. ***B***, The ability of PLFS to depotentiate SC LTP is completely blocked by 1 μM clozapine (white bar). Upper traces to the right show representative EPSPs at baseline (black lines) and 60 min following SC HFS (redlines) while lower traces show baseline (black lines) compared to 60 min following PLFS (red lines). Calibration: 1 mV, 5 ms.

Data were collected and analyzed using PClamp software (Molecular Devices). Data in the text are expressed as mean ± SEM. A two-tailed Student’s *t* test was used for comparisons between groups. Statistical comparisons were based on analysis of input-output curves at the 50% maximal point at baseline and sixty minutes following tetanic or 1-Hz stimulation, with *p* < 0.05 considered significant (Izumi and Zorumski, 1995). Figure 1*A* shows an example of this type of analysis. The time course graphs in all figures display results from continuous monitoring of EPSPs using the 50% maximal stimulus from the baseline IO curve as the 100% response. Data presented in the text are derived from analysis of IO curves as noted above. Analyses were done using commercial software (SigmaStat, Systat Software).

## Results

In prior studies, we found that 1 Hz × 15 min LFS of TA (perforant path, P) inputs to area CA1 produces only a transient depression of baseline transmission of SC synapses (Izumi and Zorumski, 2008). This same TA LFS, however, persistently depotentiates previously established LTP in the SC pathway when TA stimulation is administered an hour or so following induction of stable LTP (Fig. 1*A*; 143.0 ± 7.0% change in EPSP slope 60 min following HFS vs 80.7 ± 8.5% change 60 min following TA LFS, *p* = 0.0001, *N* = 7). Because prior studies have shown that activation of D4Rs are involved in homosynaptic LTP-D and NRG1-mediated chemical depotentiation of SC LTP at short intervals following LTP induction (Kwon et al., 2008), we were interested in determining whether D4Rs also contribute to TA-induced SC LTP-D an hour or more following LTP onset. To test this, we first examined the effects of clozapine, an antipsychotic drug that inhibits D2-like DA receptors with higher affinity for D4Rs (Sanyal and Van Tol, 1997). We found that 1 μM clozapine did not alter the transient synaptic depression observed during 1-Hz TA stimulation but completely blocked the ability of this stimulation to persistently depotentiate SC synapses (143.7 ± 10.5% change in EPSP slope 60 min following HFS vs 139.8 ± 11.9% change 60 min following TA LFS, *p* = 0.507, *N* = 5; Fig. 1*B*).

**Figure 2.**
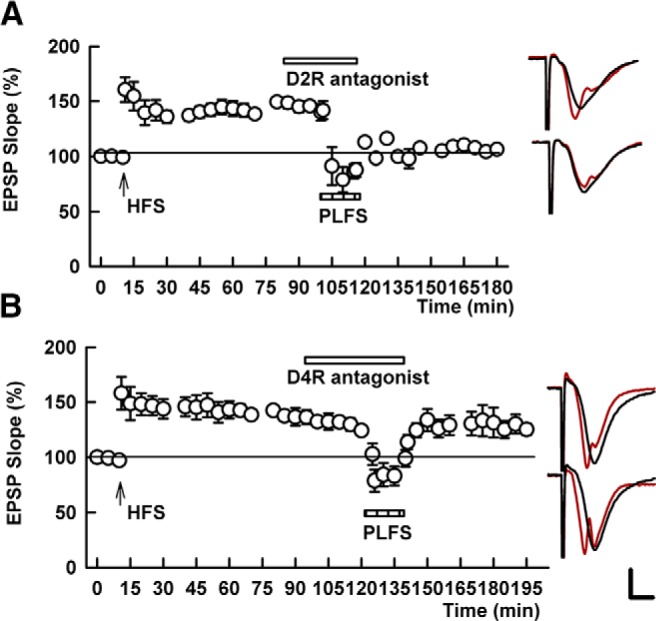
TA-induced LTP-D involves D4Rs. ***A***, The graph shows the inability of a selective D2R antagonist (0.2 μM L-741,626) to block PLFS induced depotentiation of SC LTP. SC HFS was delivered at the arrow; PLFS was administered during the hatched bar. ***B***, In contrast, a selective D4R antagonist (0.1 μM L-745,870) completely inhibited depotentiation. Traces to the right show representative EPSPs as in Figure 1. Calibration: 1 mV, 5 ms.

**Figure 3.**
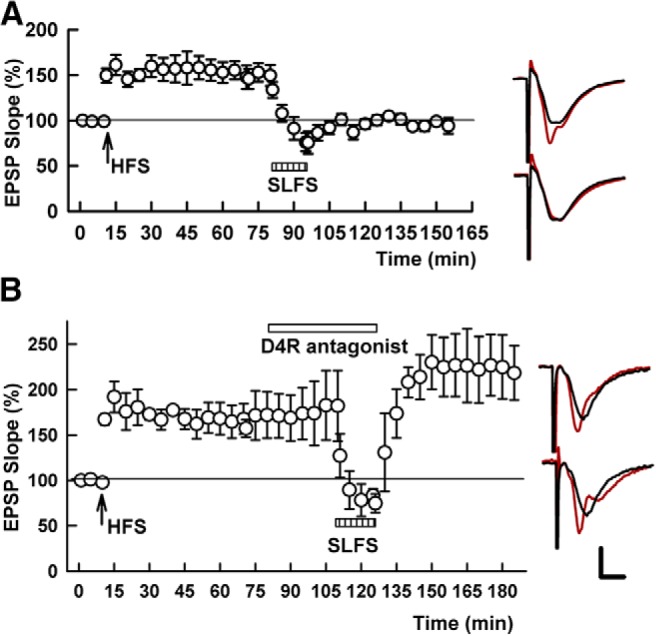
A D4R antagonist blocks homosynaptic SC depotentiation. ***A***, The graph shows the ability of SC LFS [SLFS (1 Hz × 15 min), hatched bar] to depotentiate previously established SC LTP. SC HFS was administered at the arrow. ***B***, The D4R antagonist, 0.1 μM L-745,870, blocked homosynaptic SC depotentiation. For reasons that are uncertain, we observed an increase in variance of EPSPs during perfusion of the D4R antagonist in this set of studies but not in the studies shown in Figure 2. Traces to the right show representative EPSPs as in Figure 1. Calibration: 1 mV, 5 ms.

We extended observations with clozapine using more selective antagonists against D2-type receptors. Unlike clozapine, L-741,626 (0.2 µM), a selective D2 receptor antagonist (Bowery et al., 1996), had no effect on TA-induced LTP-D (134.7 ± 3.8% change in EPSP slope 60 min following HFS vs 96.0 ± 6.3% change 60 min following TA LFS, *p* = 0.0012, *N* = 5; Fig. 2*A*). In contrast, the D4R-selective antagonist, L-745,870 (Clifford and Waddington, 2000), blocked LTP-D at a concentration of 0.1 µM (134.2 ± 1.4% change in EPSP slope 60 min following HFS vs 123.0 ± 4.7% change 60 min following TA LFS, *p* = 0.306, *N* = 6; Fig. 2*B*). These results with D4R antagonists also prompted us to examine whether D4Rs contribute to homosynaptic depotentiation in the SC collateral pathway. Consistent with prior studies,we found that 1 Hz × 900 pulse LFS of SC inputs reliably depotentiated SC LTP when administered an hour or more following LTP induction (142.0 ± 15.1% 60 min after SC HFS and 96.4 ± 5.5% 60 min after SC LFS, *N* = 5, *p* = 0.022; Fig. 3*A*; Izumi and Zorumski, 1993). This homosynaptic SC LTP-D was completely blocked by 0.1 µM L-745,870 (155.9 ± 10.4% 60 min after SC HFS vs 209.0 ± 42.1% 60 min after L-745,870, *N* = 5, *p* = 0.312; Fig. 3*B*). Taken together, these results indicate that D4Rs contribute to synaptically driven SC depotentiation resulting from either heterosynaptic TA or homosynaptic SC LFS, even 1 h or more following LTP induction.

**Figure 4.**
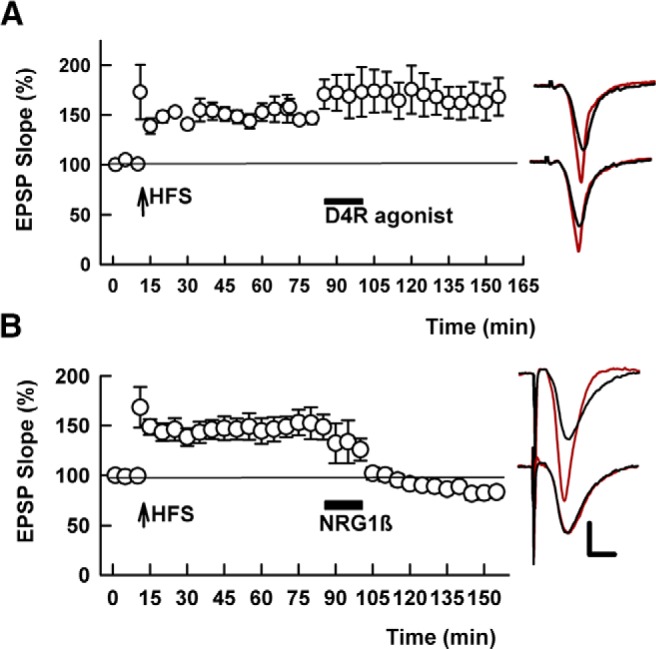
Exogenously administered NRG1β, but not a D4R agonist, depotentiates SC LTP. ***A***, A selective D4R agonist,0.2 μM PD-168,077 (black bar), failed to depotentiate SC LTP when administered for 15 min 60 min following LTP induction. SC HFS was administered at the arrow. ***B***, In contrast to the D4R agonist, 1 nM NRG1β (black bar) induced chemical depotentiation of SC LTP. Traces to the right show EPSPs as in Figure 1. Calibration: 1 mV, 5 ms.

**Figure 5.**
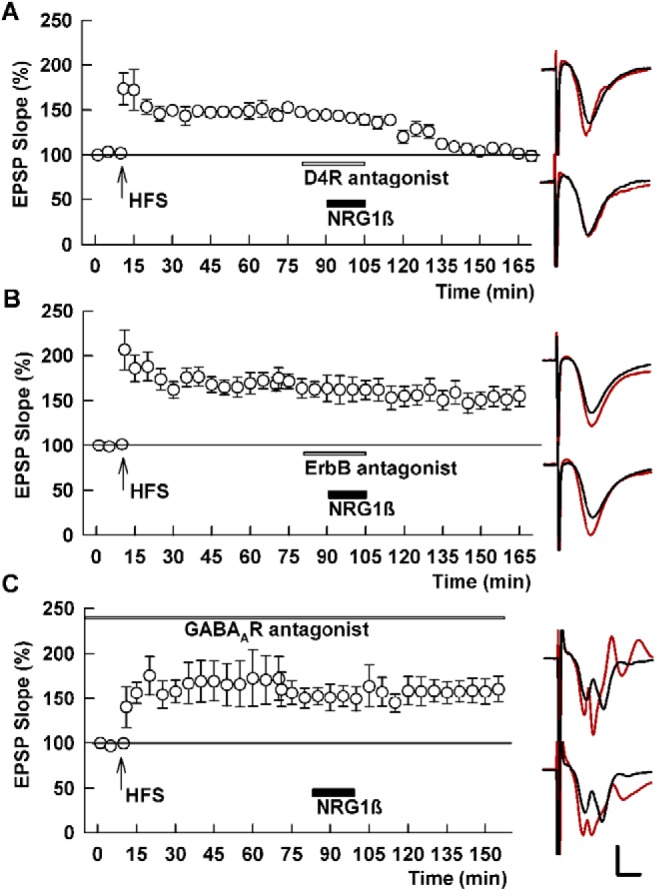
Depotentiation by NRG1β is insensitive to D4R antagonism but blocked by an ErbB antagonist and PTX. ***A***, In the presence of 0.1 μM L-745,870 (white bar), 1 nM NRG1β (black bar) induces SC depotentiation. SC HFS was administered at the arrow. ***B***, ***C***, In contrast to the D4R antagonist, a pan-ErbB antagonist (10 μM PD-158,780, white bar) blocks NRG1β-induced depotentiation (***B***), as does the GABA_A_R antagonist, 1 μM PTX (***C***). Traces show representative EPSPs as in Figure 1. Calibration: 1 mV, 5 ms.

Earlier studies indicated that a selective D4R agonist alone was able to mimic the effects of homosynaptic SC LFS and NRG1 at early time points (30 min) after LTP induction (Kwon et al., 2008) but that NRG1 itself was ineffective when administered at later time points after LTP induction (60 min; Kwon et al., 2005).We found that the selective D4R agonist, PD-168,077 had no effect on LTP when administered alone an hour or more following SC LTP induction at either 0.2 μM (148.1 ± 6.7% 60 min after SC HFS vs 141.8 ± 9.1% 60 min after PD-168,077, *p* = 0.581, *N* = 5; Fig. 4*A*; Kwon et al., 2008) or 10 µM (138.2 ± 6.0% 60 min after SC HFS vs 132.2 ± 8.2% after PD-168,077, *N* = 5, *p* = 0.409). We did find, however, that administration of 1 nM NRG1β for 15 min depotentiated SC LTP when administered 60 min following LTP induction (140.5 ± 7.8% of baseline 60 min following HFS vs 96.2 ± 7.8% following NRG1β, *p* = 0.002, *N* = 6; Fig. 4*B*). NRG1β-induced depotentiation was not inhibited by the D4R antagonist (149.9 ± 2.8% 60 min following HFS vs 91.4 ± 5.9% after NRG1β + L-745,870, *p* < 0.0001, *N* = 5; Fig. 5*A*), indicating that NRG1β-induced LTP-D does not require D4R activation at late time points after LTP induction.

NRG1β-induced depotentiation was blocked by 10 μM PD-158,780, a pan ErbB antagonist (133.4 ± 3.9% after HFS vs 128.5 ± 2.3% after NRG1β, *p* = 0.130, *N* = 5; Fig. 5*B*) and by 0.1 μM AG-1478, a more selective ErbB4 antagonist (158.7 ± 7.5% vs 149.0 ± 10.5%, *p* = 0.474, *N* = 5; Li et al., 2007). Because earlier studies indicated that activation of ErbB4 can stimulate GABA release from interneurons (Woo et al., 2007) and we previously found a role for GABA_A_Rs in TA-induced LTP-D (Izumi and Zorumski, 2016), we examined the effects of PTX, a GABA_A_R antagonist, on chemical depotentiation by NRG1β. We found that administration of 1 μM PTX overcame the effects of NRG1β on previously established SC LTP (150.3 ± 15.2% after HFS vs 133.9 ± 3.7% after NRG1β + PTX, *p* = 0.235, *N* = 5; Fig. 5*C*).

**Figure 6.**
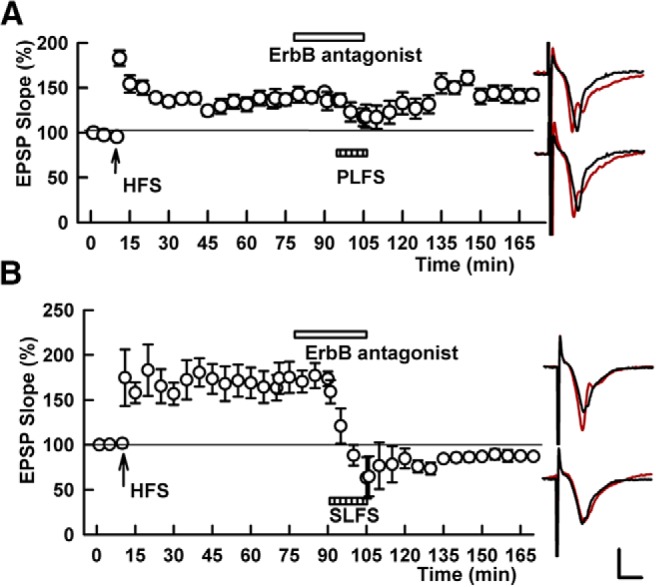
An ErbB antagonist blocks TA-induced, but not homosynaptic SC depotentiation. ***A***, In the presence of 10 μM PD-158,780, TA stimulation (PLFS, hatched bar) fails to induce persistent SC depotentiation. SC HFS was administered at the arrow. ***B***, In contrast, the ErbB antagonist fails to block depotentiation induced by homosynaptic SC LFS (SLFS, hatched bar). Traces show representative EPSPs as in Figure 1. Calibration: 1 mV, 5 ms.

The ErbB antagonist, PD-158,780, also blocked TA-induced LTP-D (135.7 ± 11.1% of baseline 60 min following HFS vs 136.6 ± 7.2% following TA LFS, *p* = 0.916, *N* = 5; Fig. 6*A*), but did not alter homosynaptic SC depotentiation (144.9 ± 6.1% after HFS vs 97.4 ± 5.1% after SC LFS, *N* = 5, *p* = 0.0003, Fig. 6*B*). These findings suggest common mechanisms in NRG-induced and TA-induced LTP-D, but not homosynaptic LTP-D.

**Figure 7.**
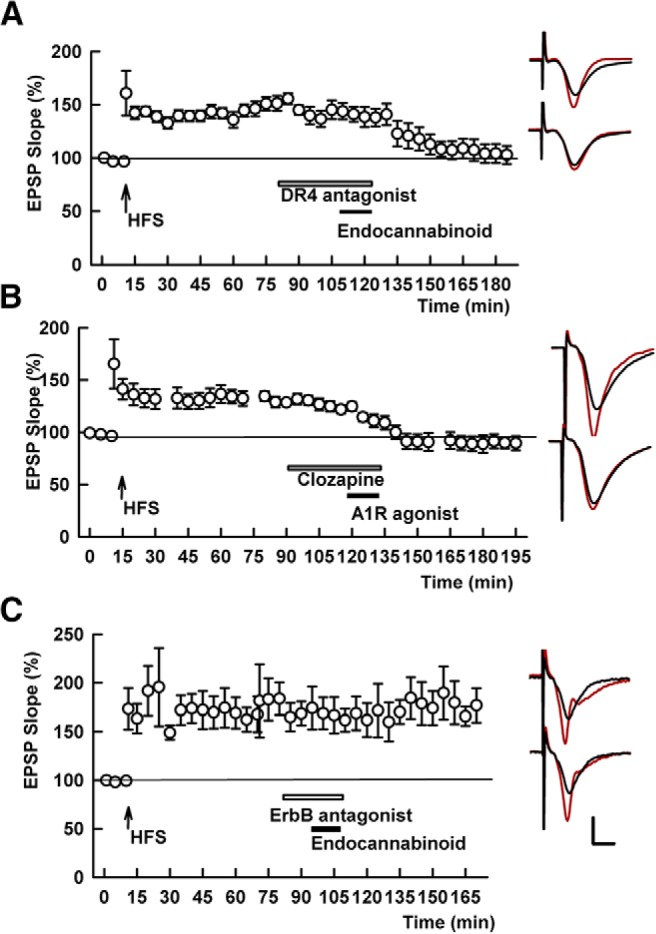
D4R antagonism does not block chemical depotentiation by CB1R or adenosine A1R activation, but ErbB antagonism blocks the effects of a CB1R agonist. ***A***, In the presence of the D4R antagonist, L-745,870 (white bar), the endocannabinoid, 20 μM 2-AG (black bar), reversed SC LTP. SC HFS was administered at the arrow. ***B***, Similarly, clozapine, a DAR antagonist with selectivity for D4Rs (white bar), failed to block depotentiation by 10 nM CPA, a selective A1R agonist (black bar). ***C***, The effects of 2AG on SC STP were blocked by the ErbB antagonist, PD-158,780. Traces show representative EPSPs as in Figure 1. Calibration: 1 mV, 5 ms.

In our prior studies of TA-induced LTP-D, we found that activation of endocannabinoid CB1Rs and adenosine A1Rs contribute to the cascade of events leading to synaptic resetting, with CB1Rs involved earlier in the pathway than A1Rs (Izumi and Zorumski, 2008). These observations prompted us to examine whether D4R blockade alters the effects of pharmacological activation of either CB1R or A1Rs on SC LTP. We found that the endocannabinoid agonist, 2-arachidonoylglycerol (2-AG, 20 μM) depotentiated SC LTP in the presence of the D4R antagonist, L-745,870 (141.5 ± 7.9% of baseline 60 min after HFS vs 105.6 ± 3.7% after 2AG, *p* = 0.0034, *N* = 5; Fig. 7*A*). Similarly, in the presence of 1 µM clozapine, 10 nM cyclopentyladenosine (CPA), an A1R agonist, readily reversed SC LTP (136.7 ± 4.2% of baseline 60 min after HFS vs 82.3 ± 5.4% after CPA, *p* = 0.0004, *N* = 5; Fig. 7*B*). Interestingly and unlike the D4R antagonist, the ErbB antagonist, PD-158,780, completely blocked 2AG-mediated depotentiation (156.4 ± 16.9% of baseline 60 min following HFS vs 159.5 ± 13.4% 60 min after 2AG, *p* = 0.542, *N* = 5; Fig. 7*C*). Taken together with our prior studies (Izumi and Zorumski, 2008, 2016), these results indicate that both CB1R and A1R activation likely occur downstream of D4R activation and that ErbB receptor activation occurs downstream of CB1Rs but upstream of A1Rs.

## Discussion

Hebbian plasticity in the hippocampus is thought to play a key role in learning and memory (Kandel et al., 2014; Nicoll, 2017), but has the limitation that this type of use-dependent synaptic change is saturable unless there are mechanisms by which synapses can be reset for future plasticity and learning (Turrigiano, 2011). Hence, there has been interest in understanding mechanisms by which synapses depotentiate following LTP induction, including homeostatic changes, and forms of homosynaptic and heterosynaptic depotentiation. We previously found that LFS of direct TA (perforant path) inputs to area CA1 can depotentiate previously established SC pathway LTP, without having persisting effects either on baseline transmission or the ability of subsequent stimulation to induce LTP or LTD at SC synapses (Izumi and Zorumski, 2008). TA-induced SC depotentiation involves complex signaling mechanisms including GABA_A_Rs, CB1Rs and adenosine A1Rs along with activation of two MAP kinases, ERK1/2 and p38 MAPK (Izumi and Zorumski, 2008, 2016).Surprisingly, this form of synaptic plasticity does not appear to involve activation of glutamate receptors (Izumi and Zorumski, 2016). In the present work, we provide evidence that TA-induced SC depotentiationinvolves activation of D4 type DA receptors and ErbB signaling.

Prior studies indicate that DA plays a key role in modulating hippocampal function, including long-term forms of synaptic plasticity (Furth et al., 2013). Intriguingly, DA innervation of area CA1, particularly afferents arising from DA cell bodies in the ventral tegmental area to SLM, is relatively sparse, with an absence of DA transporters but significant expression of DA receptors in SLM (Kwon et al., 2008; Smith and Greene, 2012; Kempadoo et al., 2016). Other work indicates that adrenergic terminals in the hippocampus can release DA and that afferents from the locus coeruleus may be critical for providing DA signals to the CA1 region (Smith and Greene, 2012; Kempadoo et al., 2016). Additionally, norepinephrine can directly activate D4Rs providing another way that the adrenergic system can stimulate DAreceptors to modulate CA1 function (Root et al., 2015; Sánchez-Soto et al., 2016). If norepinephrine is the key monoamine transmitter driving TA-induced SC depotentiation, our present results indicate that its effects on SC LTP involve D4Rs. We previously found that exogenous norepinephrine, acting at adrenergic receptors, prevented rather than promoted homosynaptic SC depotentiation (Katsuki et al., 1997), indicating that norepinephrine alone does not mimic the effects of TA stimulation.

Buonanno and colleagues previously demonstrated a role for D4Rs in a form of chemical depotentiation of SC LTP induced by NRG1β and involving activation of ErbB receptors (Kwon et al., 2005, 2008). They also found that NRG1β promoted release of DA in the CA1 region, indicating that the effects of NRG and DA are intertwined. D4Rs also contributed to homosynaptic SC depotentiation following theta pulse stimulation administered shortlyafter LTP was induced (Kwon et al., 2008).These forms of depotentiation, particularly NRG1β-induced LTP-D, were observed within 30 min of LTP induction, but not 50 or more min after stable LTP had been established. Prior studies have found that LTP is more readily reversed early (<30 min) after induction rather than later after induction (an hour or more; Izumi and Zorumski, 1993; Barr et al., 1995; Stäubli and Chun, 1996; Kwon et al., 2005, 2008). Several factors may contribute to the stability of LTP over time and the ease with which LTP can be reversed, including the stimuli used to induce LTP (e.g., single vs multiple HFS), the conditions under which LTP was induced (e.g., ionic conditions, age of animals; Huang et al., 1996; Huang and Kandel, 2005) and the type and duration of stimulation used to induce depotentiation (briefer HFS trains vs LFS of varying durations; Fujii et al., 1991; Izumi and Zorumski, 1993; Bashir and Collingridge,1994; Wagner and Alger, 1995; Kwon et al., 2005). In our studies we used a single 100 Hz × 1 s HFS to induce LTP that remained stable for over an hour in P30 rat hippocampal slices and reversed this LTP using 15 min 1-Hz LFS; the LFS used for depotentiation was selected based on a standard LFS that has been used to induce homosynaptic LTD (Dudek and Bear, 1992) or LTP-D in the SC pathway (Izumi and Zorumski, 1993). These differences in stimulation paradigms may contribute to the fact we were able to reverse SC LTP an hour or more after induction.

In the hippocampus, ErbB4 receptors are a predominant NRG receptor (Li et al., 2007) and are expressed on GABAergic interneurons (Vullhorst et al., 2009; Neddens and Buonanno, 2010; Bean et al., 2014). NRG1β disinhibits interneurons via ErbB4 and promotes release of GABA (Woo et al.2007). Consistent with this, we found that NRG1β-induced SC depotentiation was blocked by a GABA_A_R antagonist, suggesting that NRG1-induced GABA release may be critical for this form of synaptic resetting. The effects of NRG1β on stable LTP were not reversed by a D4R antagonist, suggesting that mechanisms contributing to early and later LTP reversal by NRG1β and D4R activation likely differ. Other evidence indicates that D4Rs, like ErbB4, are expressed on some interneurons (Mrzljak et al., 1996), and recent work has shown that low DA release evokes feedforward inhibition in CA1 that is mediated by D4Rs on parvalbumin (PV)-positive interneurons (Rosen et al., 2015). Despite the fact that TA-induced LTP-D is blocked by a D4R antagonist, we found that a D4R agonist alone failed to depotentiate SC LTP when administered 1 h after LTP induction, although NRG1β was effective. At present we do not know which interneurons contribute to TA-induced SC depotentiation, but note that several types of interneurons have dendrites in or extending to SLM, including PV+ interneurons (axo-axonic and some basket cells), cholecystokinin-positive interneurons and neurogliaform cells (Klausberger et al., 2003; Klausberger and Somogyi, 2008; Klausberger, 2009; Overstreet-Wadiche and McBain, 2015) along with longer-range GABAergic inputs from entorhinal cortex to SLM (Basu et al., 2016).

**Figure 8.**
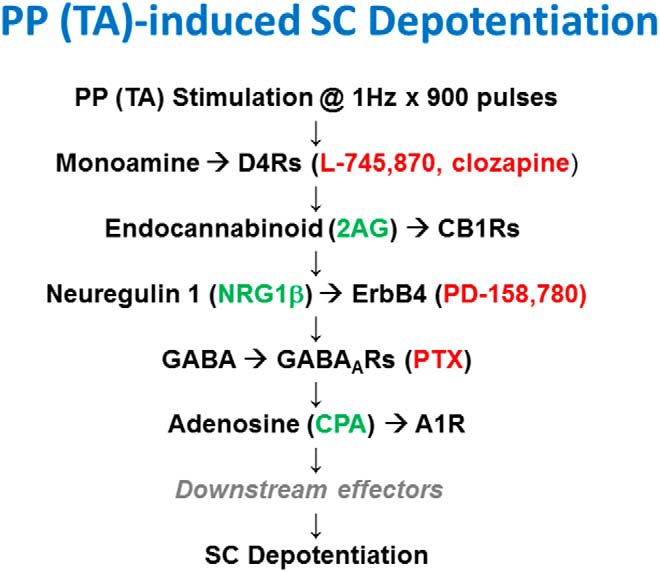
The diagram depicts a current scheme for TA-induced SC LTP-D based on prior studies (Izumi and Zorumski, 2008, 2016) and the present results. Agents that promote chemical depotentiation are shown in green while agents that inhibit TA-induced SC depotentiation are shown in red.

Based on the effects of selective agonists and antagonists, we have proposed a scheme for TA-induced SC depotentiation in which activation of ERK1/2, diacylglycerol lipase, and endocannabinoid synthesis are involved relatively early in the cascade, with activation of GABA_A_Rs, p38 MAPK, and A1Rs participating as more downstream effectors (Izumi and Zorumski, 2016). Our present results indicate that D4Rs act more proximally in the depotentiation scheme than endocannabinoids, NRG1 or adenosine. D4Rs can also stimulate ERK1/2 (Bitner et al., 2006; Oak et al., 2001), which we have found is involved relatively early in the depotentiation scheme (Fig. 8). Consistent with Kwon et al. (2005, 2008), we also found that a D4R agonist alone was not capable of inducing depotentiation when administered an hour or more after LTP had been established. These findings suggest that D4R activation is necessary but not sufficient to induce TA-mediated synaptic resetting, and that D4Rs and ErbB receptors act independently in the cascade. Based on experiments to date, adenosine is the most distal signal in the cascade yet identified. We have tested some messengers linked to A1R-induced LTD (Brust et al., 2006; Chen et al., 2014) in our prior work and found a role for p38 MAPK (Izumi and Zorumski, 2016). However, in our experiments p38 MAPK appeared to act upstream of A1Rs and did not block the effects of chemical depotentiation by CPA.

The involvement of D4Rsand NRG1β in TA-induced SC depotentiation provides a possible link to the role of the DA and NRG systems in cognitive defects associated with psychiatric illnesses such as schizophrenia, major depression, substance use disorders, and attention deficit hyperactivity disorder (Buonanno, 2010; Shamir et al., 2012; Penzes et al., 2013). D4Rs in the hippocampus modulate gamma oscillations that are important in attention and information processing (Andersson et al., 2012). Similarly, by altering the ability of synapses to reset following LTP, changes in DA and NRG function could be important in driving changes in hippocampal input-output relationships observed in animal models of psychiatric illnesses (Airan et al., 2007), and perhaps in the ability to learn and remember new information. The net effects of changes in DA and NRG modulation, even within the CA1 region, likely depend on input-specific actions and the subtypes of receptors that are stimulated, as well as on the state of glutamate synapses at the time of modulator release. In *Drosophila*, DA plays complex roles in memory and is required for both learning and forgetting (Berry et al., 2012); furthermore, sleep has been found to promote memory in *Drosophila* by impairing DA-driven forgetting (Berry et al., 2015). From the perspective of D4Rs, hyperdopaminergic tone would be expected to promote reversal of hippocampal and cortical LTP, perhaps leading to defects in longer-term memory storage (Xu et al., 2009), while lower DA tone could result in more persisting LTP in the CA1 region, perhaps dampening the ability of synapses to reset for future potentiation and learning. Thus efforts to modulate D4Rs and NRG signaling could have unique effects in a range of neuropsychiatric illnesses depending on the state of glutamate synapses, perhaps leading to novel ways to dampen the cognitive dysfunction that underlies illness-related disability.

## Synthesis

Reviewing Editor: Pablo Castillo, Albert Einstein College of Medicine Decisions are customarily a result of the Reviewing Editor and the peer reviewers coming together and discussing their recommendations until a consensus is reached. When revisions are invited, a fact-based synthesis statement explaining their decision and outlining what is needed to prepare a revision will be listed below. The following reviewer(s) agreed to reveal their identity: Gary Lynch.

Your manuscript has been evaluated by two experts in the field. Although their opinions were initially split, the following summarizes their common views on your study. They both appreciate the potential significance of your findings, but they also raise significant concerns on the methodology, lack of key control experiments and interpretation of the results. Specifically, they highlight the following points:

- Test the effect of most experimental treatments on basal transmission; i.e. naïve pathway in a two-pathway experiment.

- Clarify how differences between I/O curves were assessed and provide some justification/rationale for using this uncommon method for calculating percent LTP.

- Provide mechanistic explanations for unexpected observations (e.g. A1R agonist not depressing synaptic transmission).

- Based on current knowledge on LTP depotentiation, a more scholar discussion of the present findings is required.

The reviewers' specific comments (see below) should be fully addressed before your study can be considered for publication.

-----------------------------------------------------------------------

REVIEWER #1

In the manuscript by Izumi and Zorumski, the authors describe experiments aimed at delving further into the mechanisms underlying depotentiation of CA1 synapses induced with low-frequency stimulation of Temperoammonic (TA) inputs. The studies may provide important insight into the actions and/or cognitive side effects of antipsychotic medications such as clozapine. Because the pathways involved likely require non-neuronal elements such as glial cells, and possibly the anatomical separation of the particular synaptic elements, the slice preparation is an appropriate way of investigating the topic. The manuscript is clearly written and the experimental design and interpretation of the data are straightforward. I only have a few minor issues that should be addressed.

Materials:

1st paragraph: "..picrotoxin was kept constant throughout the experiments..." is confusing. How about ".. picrotoxin was washed on at the initiation of the recordings and included in the ACSF for the duration of the experiments."

Drug names:

Although I see where some manufacturers refer to the drugs with commas midway through the drug names, the authors are inconsistent in doing so, even for the same drug. For example, PD158780 is also listed in several places as PD-158,780, which can be confusing at least. The authors should pick one format, preferably for all drugs if applicable, and use it throughout.

Figures:

1) Given the similarities of some of the drug names, I suggest labeling the bars on the graphs with something more generic, like "D2R antagonist", "ERBb4 antagonist", with the actual drug names and relevant drug concentrations in the figure legends. Perhaps at least for the drug names that are similar (PD's and L's).

2) I agree with the previous reviewer that the dashed lines for the traces don't look very good, and actually omit portions of the trace. Red vs. black solid lines are perfectly good (no dashes in traces please).

3) It may be an artifact of the pdf conversion process, but please check that line thicknesses match across and within figures. (Figure 3B and Figure 6 look thinner than Figures 1 and 2 and 3A, for example).

4) Again, possibly from the pdf conversion, some of the arrowheads in Figure 8 are partially gone. Best to make them bigger so that the meaning isn't changed if it happens in downloads.

5) I suggest that some more drug names be included in Figure 8. For example, add "(2AG)" in green with CB1Rs; "(CPA)" in green with A1Rs, and "clozapine" in red together with L745,870. This figure was a nice reference for the reader, so making it inclusive.

REVIEWER #2

This manuscript reports a number of interesting findings related to reversal ('depotentiation') of long-term potentiation (LTP) in the Schaffer-commissural (S-C) projections to proximal, apical dendrites of hippocampal field CA1. The authors primary concern is with an intriguing heterosynaptic depotentiation produced by stimulation of the temporoammonic projections to the outermost branches of CA1 pyramidal neurons. Many of the results involve the much studied homosynaptic depotentiation produced by manipulations of the S-C itself following induction of LTP. There are several problems with the submission.

1. Controls for treatment effects in the absence of LTP. A central requirement for the claim that a given manipulation reverses LTP is evidence that it does not affect baseline responses. Otherwise, how do we know if the observed effects reflect long-term depression superimposed on LTP. The usual control for this is to show that the experimental treatments have no effect on baseline physiology ---such demonstrations are lacking in this paper. The authors may choose to argue that i) the pertinent data are described in previous publications, or ii) the reversal of LTP persists for much longer than any acute actions of the treatment. (The paper is notable for excellent demonstrations of LTP stability.) The text does not cite earlier work on controls for most of the manipulations or make arguments for the latter point. In any event, data on non-potentiated synaptic responses are needed to make a strong case for the conclusion that LTP substrates were in fact reversed.

2. Treatments known to depress baseline physiology. Related to the above, numerous studies have shown that adenosine A1 receptor agonists and the endocannabinoid 2-AG reduce control synaptic responses by depressing transmitter release. One would expect that these rapid and pronounced effects to be superimposed on potentiated fEPSPs, as shown in published studies using adenosine, but they are not in evidence in Fig.7. The absence of a transient depression for 2-AG administered in the presence of clozapine (Fig 7B) or an ErbB antagonist (Fig 7C) will be a major surprise for the endocannabinoid field. An explanation is badly needed.

3. Post-LTP induction time course over which treatments were effective. Several papers show that LTP in S-C synapses becomes steadily more resistant to depotentiation produced by afferent activity or adenosine as time after LTP induction increases. An important feature of the authors' past and present work is demonstration of reversal, whether via the temporoammonic pathway or by 1Hz stimulation of the S-C itself, at one -hour after induction. Does this reflect their use of prolonged (15 min) stimulation and/or to the presence of multiple routes for inactivating the mechanisms that support LTP? More generally, the survey of the prior literature on depotentiation is inadequate; as a result, the present findings are not placed in an appropriate framework.

4. Hypothesis regarding sequence of events leading to depotentiation needs clarification. The authors propose that enhanced activity by interneurons followed by adenosine release is the proximal cause for depotentiation of the S-C produced by stimulation of the temporoammonic pathway. Are these events occurring in the terminal field of the TA (stratum lacunosum/moleculare) or in the dendritic field innervated by the S-C (stratum radiatum)? If the latter (which seems more likely), then questions arise about how the TA related events in distal dendrites connect with GABAergic cells in more proximal dendrites. Moreover, are mechanisms shown in literature to mediate adenosine related depotentiation elicited by stimulation of the S-C also involved in heterosynaptic reversal of LTP?

5. Procedures for measuring percent LTP. The authors follow an unusual procedure involving input/out curves for calculating percent potentiation. The authors do not explain why this methodology is preferred to the more standard comparison of baseline responses to those at some point after LTP induction. In any case, a figure is needed to illustrate the method along with an explanation of how t-tests were used to compare two series of data points.
